# The *Arabidopsis thaliana* Class II Formin FH13 Modulates Pollen Tube Growth

**DOI:** 10.3389/fpls.2021.599961

**Published:** 2021-02-18

**Authors:** Eva Kollárová, Anežka Baquero Forero, Fatima Cvrčková

**Affiliations:** Department of Experimental Plant Biology, Faculty of Science, Charles University, Prague, Czechia

**Keywords:** *Arabidopsis thaliana*, At5g58160, Class II formin, pollen tube, tip growth

## Abstract

Formins are a large, evolutionarily conserved family of actin-nucleating proteins with additional roles in regulating microfilament, microtubule, and membrane dynamics. Angiosperm formins, expressed in both sporophytic and gametophytic tissues, can be divided into two subfamilies, Class I and Class II, each often exhibiting characteristic domain organization. Gametophytically expressed Class I formins have been documented to mediate plasma membrane-based actin assembly in pollen grains and pollen tubes, contributing to proper pollen germination and pollen tube tip growth, and a rice Class II formin, FH5/RMD, has been proposed to act as a positive regulator of pollen tube growth based on mutant phenotype and overexpression data. Here we report functional characterization of the *Arabidopsis thaliana* pollen-expressed typical Class II formin FH13 (At5g58160). Consistent with published transcriptome data, live-cell imaging in transgenic plants expressing fluorescent protein-tagged FH13 under the control of the FH13 promoter revealed expression in pollen and pollen tubes with non-homogeneous signal distribution in pollen tube cytoplasm, suggesting that this formin functions in the male gametophyte. Surprisingly, *fh13* loss of function mutations do not affect plant fertility but result in stimulation of *in vitro* pollen tube growth, while tagged FH13 overexpression inhibits pollen tube elongation. Pollen tubes of mutants expressing a fluorescent actin marker exhibited possible minor alterations of actin organization. Our results thus indicate that FH13 controls or limits pollen tube growth, or, more generally, that typical Class II formins should be understood as modulators of pollen tube elongation rather than merely components of the molecular apparatus executing tip growth.

## Introduction

The angiosperm microgametophyte (pollen) uses a specific mode of cell expansion, known as tip growth, to deliver the male gametes to the megagametophyte via a pollen tube that grows invasively through maternal sporophytic tissues. Similarly to other tip-growing cells, such as land plant root hairs, but also, e.g., moss protonemata (see Vidali and Bezanilla, 2012), pollen tube elongation relies on focused membrane turnover at the tip of a growing cellular protuberance, which requires a precise co-ordination of exo- and endocytosis (see Moscatelli and Idilli, 2009), and which is controlled, among other factors, also by specific organization of actin, involving both longitudinal microfilament bundles along the shank of the tube and a “fringe” of fine actin filaments (see Fu, 2015; Qu et al., 2015; Stephan, 2017; Zhang et al., 2018). Microtubules, although apparently not essential for tip growth itself, also contribute to endomembrane trafficking within elongating pollen tubes and to determining growth direction (see Fu, 2015). Understanding the molecular mechanisms that co-ordinate cytoskeletal, especially actin, and membrane dynamics is thus central also for grasping the cellular mechanisms of pollen tube development.

One of the many possible molecular players in pollen tube growth are the formins, or FH2 proteins—members of an evolutionarily old and diverse protein family defined by the presence of two conserved domains—the profilin-binding, proline-rich FH1 domain and the microfilament-nucleating and -capping FH2 domain. Formins engage in the regulation of actin nucleation and other aspects of actin dynamics, but also in microfilament- microtubule co-ordination (see e.g., Bartolini and Gundersen, 2010; Henty-Ridilla et al., 2016). Angiosperm plants possess two formin clades, termed Class I and Class II (Deeks et al., 2002), each characterized by a typical, or canonical, domain organization, although some members of each clade deviate from this typical structure (Cvrčková et al., 2004; Grunt et al., 2008). Besides of the FH1 and FH2 domains, canonical plant Class I formins are transmembrane proteins with a N-terminal extracytoplasmic domain, in several cases documented to mediate transmembrane anchorage of the formin and associated cortical cytoskeleton to the cell wall, or at least plasmalemma localization (reviewed in Cvrčková, 2013; van Gisbergen and Bezanilla, 2013; see also e.g., Diao et al., 2018; Oulehlová et al., 2019). Class I formins also occur on, and sometimes dynamically relocate between, boundaries of endomembrane system compartments, including endosomes and the tonoplast (Oulehlová et al., 2019). Class II formins, on the other hand, are never transmembrane but typically contain a N-terminal membrane lipid-binding domain related to the protooncogene PTEN (Phosphatase and Tensin Homolog), followed by the calcium-binding C2 domain and the FH1/FH2 domains tandem (Grunt et al., 2008). Members of this clade bind membranes and exhibit cortical (probably endomembrane compartment) localization in the moss *Physcomitrella patens* (Vidali et al., 2009; van Gisbergen et al., 2012). A rice canonical Class II formin, FH5, encoded by the RMD (Rice Morphology Determinant)/BUI1 (Bent Uppermost Internode 1) gene (Yang et al., 2011; Zhang et al., 2011), localizes to the surface of plastids suggesting plastid envelope interaction (Zhang et al., 2011). Related to its role in amyloplast– cytoskeleton connection, this protein also participates in gravity sensing (Song et al., 2019). Two typical *Arabidopsis* thaliana Class II formins, FH13 (At5g58160) and FH14 (At1g31810), partly co-localize with the endoplasmic reticulum and microtubules when heterologously expressed in tobacco leaf epidermis (Kollárová et al., 2020), again consistent with these proteins associating with endomembranes. Thus, formin-membrane interaction is widespread and, in some cases, involves also membranes other than the plasmalemma.

Research into plant formin’s role in tip-growth focused so far mainly on Class I members. Ectopic expression of the *Arabidopsis* formin FH1, which is naturally expressed mainly in vegetative tissues, results in excessive formation of thick actin cables at the expense of the fine actin fringe and to pollen tube tip swelling (Cheung and Wu, 2004). Naturally pollen-expressed *Arabidopsis* FH3 and FH5 formins contribute to the organization of the actin fringe; their loss leads to defects of subapical actin structure that results in pollen tube thickening and wavy or kinky tube growth (Cheung et al., 2010; Lan et al., 2018). Overexpression of a deletion derivative of FH3 in pollen leads to similar defects as that of FH1—i.e., excessive actin cable formation and tip swelling—while FH3 downregulation by RNAi inhibits tube elongation (Ye et al., 2009). *Arabidopsis* FH5 has been also documented to nucleate actin on membrane vesicles during pollen germination, contributing to tip growth initiation (Liu et al., 2018). Also the lily class I formin LlFH1 localizes to exocytotic vesicles associated with the leading edge of the actin fringe (Li et al., 2017). Findings from pollen are corroborated also by reports from another tip-growing cell type—the root hairs—where overexpression of *Arabidopsis* FH4 and FH8 results in polarity loss manifesting as initiation of ectopic growing tips (Yi et al., 2005), while expression of a dominant negative FH8 allele suppresses tip growth (Deeks et al., 2005). Impaired root hair growth has been reported also from rice mutants defective in the Class I formin OsFH1 (Huang et al., 2013). Although the mutant phenotypes are, as a rule, relatively mild, most likely due to functional redundancy among multiple co-expressed Class I formins, together these observations suggest a general role of Class I formins in co-ordination of exocytosis and actin remodeling at the border of the growing zone of tip-growing cells.

Typical Class II formins have also been implicated in tip growth. Rice FH5/RMD localizes to the pollen tube tip; mutants with impaired function of this gene exhibit decreased pollen germination and pollen tube elongation rates, as well as twisted and thickened pollen tubes, most likely due to altered actin organization (Li et al., 2018). In the tip-growing filamentous stage (protonema) cells of *P. patens*, Class II formins are also apically localized and required for proper cell elongation (Vidali et al., 2009; van Gisbergen et al., 2012), possibly consistent with Class II formin role analogous to that of their Class I compartments. Nevertheless, a recent report (van Gisbergen et al., 2020) proposes the intriguing possibility of a “division of labor” between Class I and Class II formins in moss protonema tip growth, with the former engaging mainly in exocytosis while the later participate in endocytotic membrane retrieval. Besides of the above-mentioned report on rice FH5, very little is known about tip growth-related roles of angiosperm Class II formins. The only *Arabidopsis* members of this clade somewhat experimentally characterized until now are FH16, reported to bind and bundle microtubules and microfilaments *in vitro* (Wang et al., 2013), FH14 and FH13. FH14 associates with microtubules and participates in cytokinesis and male gametogenesis (Li et al., 2010), binds to actin barbed ends *in vitro* (Zhang et al., 2016), and partly co-localizes in transient heterologous expression with its relative FH13, whose actin-binding FH2 domain can form heterodimers with that of FH14 in a yeast two hybrid assay (Kollárová et al., 2020).

However, no studies addressing the role of Class II formins in mature dicot pollen have been published so far. Since FH13 is notorious for its abundant expression in *A. thaliana* pollen (Blanchoin and Staiger, 2010), and together with FH20 also in root hairs (Schoenaers et al., 2017), suggesting possible participation in tip growth, we have chosen this gene for a functional study focusing, to our knowledge for the first time, on possible role of this Class II formin in the male gametophyte. Our mutant observations and overexpression experiments suggest that, surprisingly, FH13 acts as a negative modulator of pollen tube growth, hinting at a novel aspect of the functional diversity of plant formins.

## Materials and Methods

### Bioinformatics

For phylogenetic analysis, a collection of typical Class II formin sequences from *A. thaliana* and 11 additional angiosperm species has been established by exhaustive BLAST searches of the RefSeq section of GenBank after appropriate taxonomic restriction of the database using *A. thaliana* FH13, FH14, FH18, and FH20 as queries. Only sequences exhibiting the canonical Class II domain layout consisting of PTEN, C2, FH1, and FH2 domains were collected. For some genes, multiple predicted splicing products have been obtained; in such cases, variants best corresponding to the canonical Class II domain organization have been used for further analyses. Each sequence has been assigned a label reflecting its closest *Arabidopsis* relative as inferred from reverse BLAST searches of the *A. thaliana* proteome using the non-*Arabidopsis* sequence as a query, leading in several cases to re-naming sequences described in our previous work (Grunt et al., 2008). Full list of sequences included in the phylogenetic analysis is provided in Supplementary Table S1.

Multiple sequence alignment was constructed, manually cleaned of unreliably aligned segments (including the highly variable and repetitive FH1 domain) and used to compute a maximum likelihood phylogenetic tree as described previously (Kollárová et al., 2020).

For species with publicly available transcriptome data collections either at the ePlant (Waese et al., 2017) or Genevestigator (RRID: SCR_002358; Hruz et al., 2008) resources, or in the Gene Expression Omnibus (GEO, RRID: SCR_016569) database (Clough and Barrett, 2016), above-ground tissues or organs with highest expression were identified and average expression level values for leaves and for mature pollen (or, in the absence of pollen data, for pollen-containing organs—anthers, flowers or male inflorescences) were obtained (see Supplementary Table S1). Pollen to leaf transcript ratios were calculated from these values.

### Plant Materials and Growth Conditions

The *A. thaliana* T-DNA insertional lines *fh13-1* (SALK_064291C) and the *fh13-2* (SALK_035314), both in the Columbia-0 (Col-0) background, were obtained from The Nottingham Arabidopsis Stock Centre (NASC, RRID: SCR_004576) and crossed with wild type (WT) Col-0 plants using standard techniques. The mutant alleles were genotyped by PCR using primers LBb1.3, FH13_291_RP_new and FH13_291_LP for the *fh13-1* allele; and SALK LBb1.3, FH13_314_LP, and FH13_314_RP for the *fh13-2* allele (for primer sequences see Supplementary Table S2; gene-specific primers were designed in online T-DNA Primer Design Tool)^1^. As WT controls in subsequent experiments, we used plants carrying the WT *FH13* allele selected from the progeny of heterozygous *fh13-1/FH13* parents. For overexpression experiments, *rdr6-12* plants (Peragine et al., 2004; Oulehlová et al., 2019) have been used. A transgenic line carrying LifeAct-GFP (Cvrčková and Oulehlová, 2017) was crossed to *fh13-1* mutant line. Homozygotes *fh13-1* and control WT *FH13* plants expressing LifeAct-GFP fusion protein were recovered from the F2 population.

Plants for crossing, propagation and pollen production were grown on Jiffy Peat Pellets in a culture chamber at 22°C under long day (16 h light/8 h dark) conditions. For experiments involving analyses of seedlings or for T2 or later transgenic plant selection, seeds were sterilized, placed on standard 1/2 MS medium (1% sucrose, 1.6% plant agar, with selection agent added as required) vertical plates and grown under the same conditions. Selected transgenic seedlings were transferred to the Jiffy Peat Pellets after 10 days and further grown as described above.

### Segregation Ratio Analyses

Homozygous *fh13-1* or *fh13-2* mutants were crossed with WT (Col-0) plants. Segregation ratio analysis was performed in two biological repeats with similar results, each involving approximately 100 plants from the F2 progeny. Insertion was detected by PCR genotyping with specific primer pairs used for genotyping (see above and Supplementary Table S2).

### RNA Isolation and Transcript Detection

To estimate expression level of the *FH13* transcript, total RNA was extracted from the tissues of interest, i.e., flower buds, open flowers, shoots 23 days after germination (DAG), 23 DAG roots, or 7 DAG seedlings of the required genotype using the RNeasy kit (Qiagen). Genomic DNA was removed from samples by DNase I (New England Biolabs) treatment prior to reverse transcription, performed according to the manufacturer’s instructions using the Transcriptor High Fidelity cDNA Synthesis kit (Roche) with random hexamer primers from the kit and one microgram of total RNA as template. To detect 5′ and 3′ terminal fragments of the *FH13* mRNA, as well as the control ubiquitin transcript, PCR reactions were performed using primers listed in Supplementary Table S2, with 26–30 cycles as indicated in Results.

### Transgenic Plant Construction

The FH13 native promoter was cloned first, generating the pEN-pATFH13 entry clone. A 3748 bases fragment upstream from the coding sequence (CDS) of FH13 was amplified by PCR with specific primers pAtFH13_for and pAtFH13_rev (see Supplementary Table S2) and cloned into the pENTR^TM^ 5′-TOPO^®^ vector (Invitrogen) using TOPO^®^ Cloning (Invitrogen) according to manufacturer’s instructions. To obtain the pAtFH13:AtFH13-Venus expression vector, the three entry clones, pEN-pAtFH13, pEN-gAtFH13 (Kollárová et al., 2020), pEN-R2-Venus^∗^-L3 (Karimi et al., 2005) and destination vector pH7m34GW (Karimi et al., 2005) were mixed in MultiSite Gateway^®^ LR recombination reaction (Invitrogen) according to the manufacturer’s instruction. The resulted expression vector was transformed into *Agrobacterium tumefaciens* (strain GV3101) and subsequently transferred by floral dip (Clough and Bent, 1998) into WT or homozygous *fh13-1* mutant plants, followed by hygromycin (Merck) selection of T1 plants on 1/2 MS plates with 1.6% plant agar. The previously published UBQ:AtFH13-YFP (Kollárová et al., 2020) overexpression vector was transformed into the *rdr6-12* mutant line and T1 transformants were selected on 1/2 MS medium with 1.6% plant agar containing glufosinate-ammonium (PESTANAL^®^, Merck). For each construct, at least two independent insertion lines, which did not noticeably differ from each other in overall phenotype or any parameters analyzed, were obtained.

### Pollen *in vitro* Cultures

For each sample, pollen from a single flower was spread on a thin layer of solid pollen germinating medium (sPGM- 0.01% boric acid, 5 mM CaCl_2_, 5 mM KCl, 1 mM MgSO_4_, 10% sucrose, and 1.5% agarose, pH 7.7) on a microscopic slide and germinated at room temperature (20–22°C) in a closed Petri dish with wet tissue to maintain humidity. For each experiment, at least four flowers from independent plants were used. Each experiment was repeated at least twice. Before microscopic observation, a drop of liquid pollen germinating medium (lPGM, i.e., sPGM without agarose) was added to the sample and then covered with a cover slip.

### Imaging and Image Analysis

Microscopic images of pollen grains and germinated transgenic pollen tubes were obtained using the Zeiss LSM880 confocal laser scanning microscope with a Plan-Apochromat 10×/0.45 or Plan-Apochromat 20×/0.8 objective. Fluorophore was excited with the 488 nm argon laser (YFP) and detected by sensitive 32-chanell Gallium arsenide phosphide (GaAsP) spectral detector. Images of germinated pollen from mutant lines were acquired using Nikon Eclipse 90i microscope with a Plan Apo 4×/0.2 objective.

For the pollen grain area measurements, images were acquired 30 min after spreading on sPGM. Area of at least 200 pollen grains per experiment was measured using built-in functions of the Fiji software (Schindelin et al., 2012). To determine levels of the FH13-FP fusion signal in WT and *fh13-1* background, the transgenic pollen grain images were also used for quantification of fluorescence intensity using Fiji software. Measurements was performed on 30–70 pollen grains in two experiments with similar results. For pollen germination assays, images were acquired at the indicated time after spreading on sPGM. In case of transgenic plants, pollen grain area, fluorescence intensity, germination and growth rates were determined from the pollen of heterozygous individuals, with non-fluorescent (WT, *fh13-1*, *rdr6-12*) sibling grains or tubes serving as internal controls. Pollen grains with pollen tube length exceeding grain diameter were counted as germinated. For measuring pollen tube growth rate, images of the same fields were taken at 4 h and at 4 h 30 min after spreading on sPGM, length of the newly grown part of the pollen tubes was determined using the manual (curve) tracking tool in Fiji, and growth rate was calculated by dividing the new tube length by the time interval.

Subcellular localization of FH13-Venus/YFP in pollen grains and tubes and actin organization in *fh13-1* and WT in pollen tubes expressing LifeAct-GFP was documented using a spinning disc confocal microscope (SDCM) with vertical sample position, alpha Plan-Apochromat 100×/1.46 Oil immersion objective, laser lines set at 488 nm and camera PRIME-95B Back-Illuminated Scientific CMOS Camera. To quantify the FH13-FP fusion signal intensity in pollen tubes, intensity profiles were generated along linear transects of single optical sections of pollen tubes using the Fiji Plot Profile tool. To quantify filamentous actin signal in *fh13-1* and WT pollen tubes, Fiji tools have been used to position adjoining rectangular 2 μm wide regions of interest (ROIs) along a grid aligned to the pollen tube direction, with 30 such ROIs covering the first 60 μm of pollen tube length from the tip. One extra ROI outside of the pollen tube has been used for determination of background parameters. For each ROI, raw integrated density and area values have been determined, and area-normalized signal intensity was obtained by dividing raw integrated density by area. Genotype- and position-specific signal intensity values were plotted after background subtraction.

### Statistics and Data Processing

Statistical analyses were performed using One-way ANOVA with *post-hoc* Tukey HSD (Honestly Significant Difference) test employing an online R-based calculator (Vasavada, 2016) or the χ^2^ test using Microsoft Excel. Boxplots were generated using the BoxPlotR tool (RRID: SCR_015629; Spitzer et al., 2014), charts and histograms were produced in Microsoft Excel.

For presentation of gel or microscopy images, photos and videos were processed in the Fiji software, using algorithmic global adjustments of brightness, contrast and intensity or artificial coloring in a manner that did not selectively obscure or enhance any part of the image (Cvrčková, 2019).

## Results

### Canonical Class II Formins Form Three Clades With Multiple Pollen-Expressed Genes

To establish the evolutionary relationships between *Arabidopsis* FH13 and other canonical Class II formins, we performed a phylogenetic analysis of all annotated protein sequences containing the full PTEN, C2, FH1, and FH2 domain set from twelve plant species covering a wide range of angiosperm diversity (*Amborella trichopoda* for basal angiosperms, *Brachypodium distachyon*, *Oryza sativa*, *Sorghum bicolor*, and *Zea mays* for grasses as monocot representatives, *A. thaliana*, *Fragaria vesca*, *Populus trichocarpa*, *Pyrus brettschneideri*, and *Vitis vinifera* for rosid dicots, *Nicotiana tabacum* and *Solanum lycopersicum* for asterid dicots). A total of 59 formin-encoding loci have been identified (Supplementary Table S1), and 55 of them were used for phylogenetic tree construction, after excluding one tobacco, one grapevine and two maize sequences that lacked substantial portions of one or more of the conserved domains due to unreliable gene prediction or sequence gaps.

**FIGURE 1:**
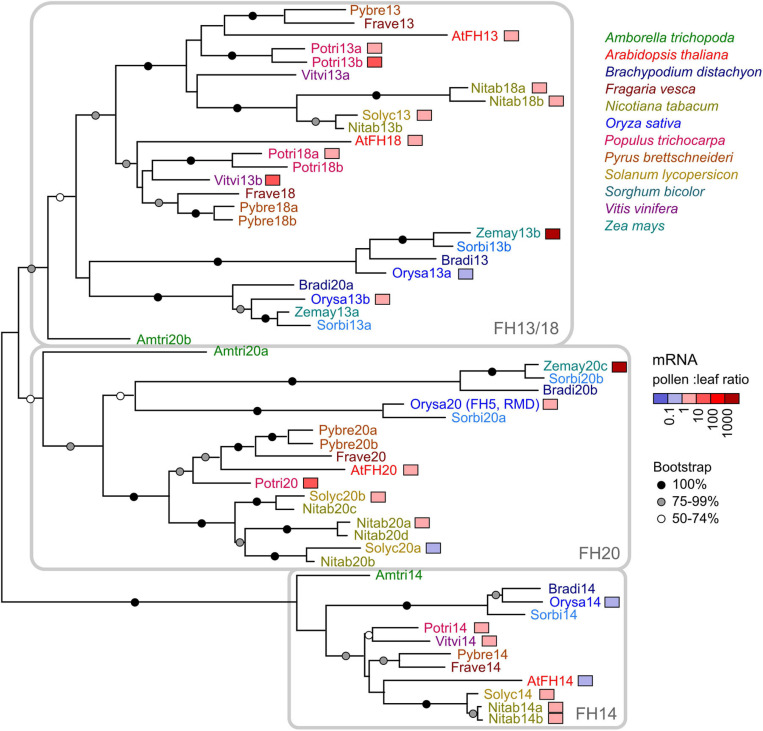
A maximum likelihood phylogenetic tree of canonical Class II formin protein sequences from 12 angiosperm species (for a list see Supplementary Table S1). *Arabidopsis* proteins are marked by the prefix “At” (thus, FH13 is shown as AtFH13). Symbols at branches denote bootstrap support (out of 500 replicates); nodes without a symbol had less than 50% support. The three clades traceable to basal angiosperms are indicated. Four fragmentary sequences are not included in the tree, namely a tobacco member of the FH13/18 clade (Nitab13a), a grapevine member of the FH20 clade (Vitvi20), and two maize FH20 clade members (Zemay20a and Zemay20b). Genes with available transcriptomic data are color-coded according to their pollen (or flower) to leaf transcript ratio.

The resulting phylogenetic tree (Figure 1) indicates the presence of three deep Class II formin clades that have separated already in the common ancestor of angiosperms (as inferred from the presence of representatives of each clade already in *Amborella*, as well as in both monocots and dicots). *Arabidopsis* FH13 and FH18 are both members of the FH13/18 clade, where independent gene duplications took place in the monocots and dicots, and, within the later, also in the asterids and rosids. The remaining two *Arabidopsis* canonical Class II formins belong to two separate clades (FH14 and FH20) well represented in all the species examined (note that rice FH5, or RMD, is a member of the FH20 clade).

For approximately half of the genes included in the phylogenetic study, transcriptomic data allowing direct comparison between the expression levels in pollen or flowers and those in leaves were available. For most of these genes including *Arabidopsis FH13*, expression levels in pollen or flowers were somewhat, but not dramatically, higher than in the leaves, although two maize, two poplar and one grapevine representatives of the FH13/18 and FH20 clades exhibited extremely high expression in pollen, anthers or male inflorescences (Figure 1 and Supplementary Table S1). Only for a few genes, including *Arabidopsis FH14*, expression in pollen or flowers was somewhat (less than by an order of magnitude—in case of *FH14* approximately by 10%) lower than that in leaves. Thus, while the documented expression patterns do not indicate widespread existence of pollen-specific canonical Class II formin paralogs, these genes, including *Arabidopsis FH13*, appear to be expressed in pollen or in (male or bisexual) flowers to an extent indicating a biologically relevant role.

### FH13 Is Widely Expressed but Not Essential for Male Gametophyte Function

**FIGURE 2:**
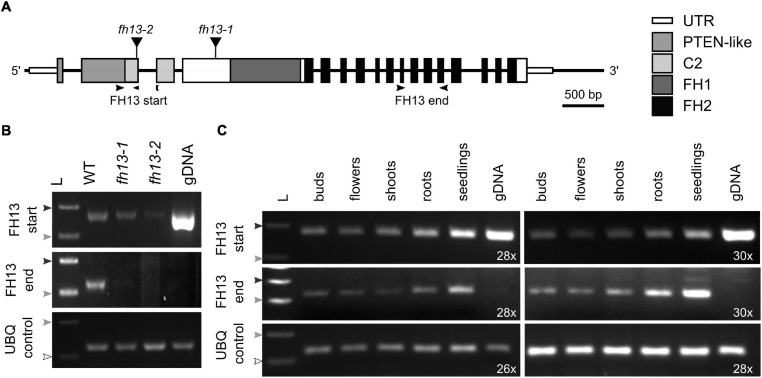
Expression of *FH13* transcripts in T-DNA insertional mutants and in various plant organs. **(A)** Schematic diagram of the *FH13* (At5g58160) locus showing location of T-DNA insertions. Narrow boxes represent untranslated regions (UTR), wide boxes represent translated exons with regions encoding conserved protein domains indicated in shades of gray or black, lines indicate non-transcribed regions and introns, black triangles show position of T-DNA insertions. Arrowheads indicate location of primers (see Supplementary Table S2) used for semiquantitative RT-PCR. **(B)** Detection of 5′ and 3′ portions of the *FH13* transcript in *fh13-1* and *fh13-2* insertional mutant and WT control 7 DAG seedlings by semiquantitative RT-PCR, with a fragment of the *UBQ* gene amplified as a control. The reactions were run for 28 cycles for *FH13* and at 24 cycles for *UBQ*. Note the presence of a strong FH13 start signal on genomic DNA, indicating that the exon junction-specific reverse primer also binds to chromosomal DNA. **(C)** Detection of 5′ and 3′ portions of the FH13 transcript in flower buds, fully opened flowers, 21 DAG roots, 21 DAG shoots, and 7 DAG seedlings of Col-0 plants by semiquantitative RT-PCR, with a fragment of the *UBQ* gene amplified as a control. The reactions were run for the indicated number of cycles. **(B,C)** The arrowheads represent DNA ladder size (white-100 bp; light gray-200 bp; dark gray-300 bp). gDNA, genomic DNA; L, DNA ladder.

In order to investigate the function of FH13 in *Arabidopsis* male gametophyte, two independent mutants of the *FH13* gene, *fh13-1* and *fh13-2*, with T-DNA insertions located in the 4rd and 2nd exon, respectively (Figure 2A), were characterized. Both heterozygous and homozygous mutant plants were viable, fertile and free of any readily noticeable phenotypic defects under standard culture conditions either *in vitro* or in a soil substrate (not shown). Semiquantitative RT-PCR analysis detected a possible presence of a partial transcript corresponding to the 5′ terminal part of the *FH13* mRNA, encoding the PTEN-like domain, expressed at a level noticeably lower than the WT transcript, in homozygous mutant *fh13-1* and *fh13-2* lines. However, this apparent transcript fragment might, in fact, be genomic DNA contamination persisting in our DNAse-treated mRNA samples, because one of the primers used, designed to span the junction of two exons, was unexpectedly found to work also on genomic DNA. Nevertheless, the 3′ end of the *FH13* mRNA, encoding the FH2 domain, was detectable only in WT plants but not in mutants, without any primer specificity issues, and we can therefore conclude that neither mutant expresses the complete gene product (Figure 2B).

To gain insight into the expression pattern of *FH13*, we performed semiquantitative RT-PCR on total RNA from a variety of WT *Arabidopsis* developmental stages and organs, namely seedlings, shoots, roots, buds and open flowers (which included pollen). *FH13* transcripts were abundantly present in all developmental stages (Figure 2C); thus, while the gene is expressed in a wide variety of tissues, including those of flowers, it cannot be considered pollen-specific, in agreement with our above-described analysis of published transcriptome data.

**FIGURE 3:**
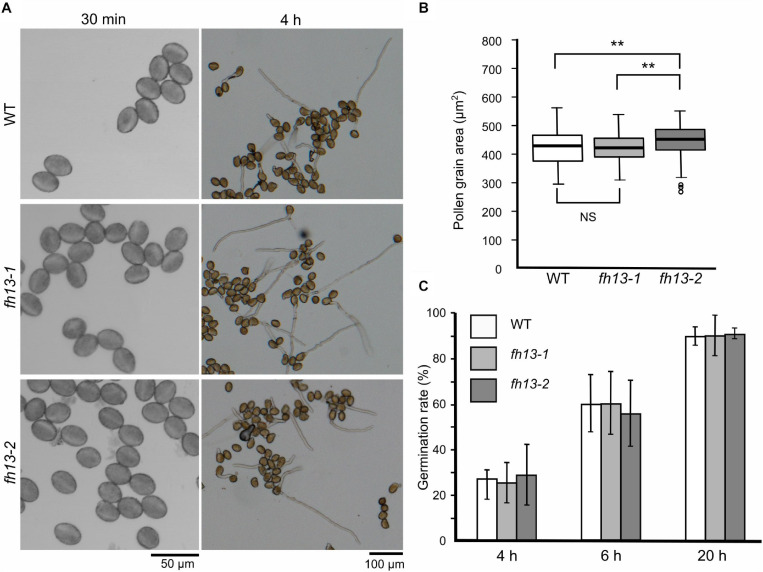
Pollen grain size and germination rate of *fh13* mutants. **(A)** Representative confocal single bright field optical sections of pollen grains at 30 min after plating on solid medium (left) and wide field images of germinated pollen after 4 h of *in vitro* cultivation (right). **(B)** Mutant and WT pollen grain area, measured 30 min after plating on solid medium (*n* > 200). Asterisks indicate statistical significance of differences (one-way ANOVA, Tukey test, ** for *p* < 0.01, NS for non-significant, *p* > 0.05). **(C)** Fraction of germinated pollen grains after the indicated time of *in vitro* cultivation. Error bars represent SD from three technical replicates. Between-genotype differences at each of the time points were non-significant (χ^2^ test, *p* > 0.05).

Pollen grains from plants homozygous for either of the two *fh13* alleles were normal in appearance and capable of germination *in vitro* (Figure 3A). While *fh13-1* pollen grain size did not differ from the WT, *fh13-2* mutants showed a small but statistically significant increase in pollen grain size (Figure 3B) compared to the WT, although biological significance of this difference is not clear. Quantification of the time course of *in vitro* pollen germination revealed no statistically significant difference between WT and *fh13* mutant pollen over 20 h of culture (Figure 3C).

Full functionality of *fh13* mutant pollen was additionally documented by segregation ratio analyses of the F2 progeny of self-fertilized heterozygous *FH13/fh13-1* or *FH13/fh13-2* plants, which did not significantly deviate from the predicted Mendelian segregation ratio (Table 1).

**TABLE 1:** Segregation of the *fh13-1* or *fh13-2* T-DNA insertion alleles in the progeny of self-pollinated heterozygous plants.

**Parent**	**WT**	***fh13* heterozygote**	***fh13* homozygote**	***P*-value**
*FH13*/*fh13-1*	17 (0.75)	53 (2.35)	20 (0.90)	0.218
*FH13*/*fh13-2*	19 (0.73)	53 (2.04)	32 (1.23)	0.193

**Parental genotypes and numbers of F2 plants of the indicated genotypes are shown (with Mendelian fractions in brackets). The observed genotype frequencies do not differ significantly from the expected 1:2.1 segregation ratio (χ^2^ test P-values are shown).**

### Mutant *fh13* Pollen Tubes Grow Faster Than WT Ones

**FIGURE 4:**
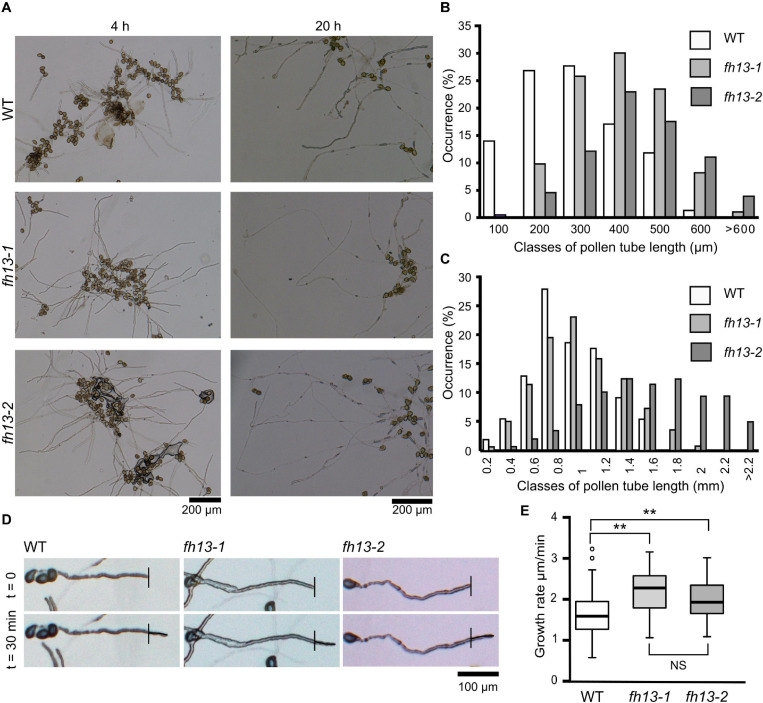
Pollen tubes of *fh13* mutants grow faster than WT *in vitro*. **(A)** Representative wide field images of WT, *fh13-1* and *fh13-2* pollen after 4 h (left) and 20 h (right) of *in vitro* cultivation. **(B,C)** Length distribution of WT, *fh13-1*, and *fh13-2* pollen tubes (*n* > 150): **(B)** after 4 h *in vitro* cultivation, **(C)** after 20 h *in vitro* cultivation. **(D)** Representative single bright field optical sections of pollen tubes taken at a 30 min interval 4 h after sowing on solid medium. Note the small but noticeable difference in the length of newly grown pollen tube (marked) between WT and the mutants. **(E)** Growth rates of WT, *fh13-1*, and *fh13-2* pollen tubes (*n* > 40), estimated from measurements of new pollen tube growth, as shown in **(D)**. Asterisks indicate statistical significance of differences (one-way ANOVA, Tukey test, ** for *p* < 0.01, NS for non-significant, *p* > 0.05).

In the pollen germination assays, we noticed that *fh13* mutant pollen produces noticeably longer pollen tubes than WT (Figure 4A, see also Figure 3A). This was also confirmed for both *fh13-1* and *fh13-2* by quantitative measurements of pollen tube length (Figures 4B,C). Already after 4 h of *in vitro* pollen culture, mean length of pollen tubes from *fh13* mutant lines was substantially greater than that of the WT control (Figure 4B), and the difference between WT and mutant pollen tubes further increased after 20 h of cultivation (Figure 4C).

Since the timing of pollen germination is not affected by the *fh13* loss of function mutations, we suspected that the observed pollen tube length difference is caused by a difference in the pollen tube growth rate, as suggested also by observation of pollen tube tips over the interval of 30 min (Figure 4D). The growth rate difference was confirmed by quantitative measurements, which showed that pollen tubes carrying either of the *fh13* mutant alleles grow faster than WT ones (Figure 4E). Taken together, these results suggest that FH13 negatively affects pollen tube growth.

### Fluorescent Protein-Tagged FH13 Is Biologically Active

Next, we generated a collection of stable transgenic *Arabidopsis* lines expressing C-terminally fluorescent protein-tagged derivatives of FH13 in order to study the protein’s intracellular localization.

**FIGURE 5:**
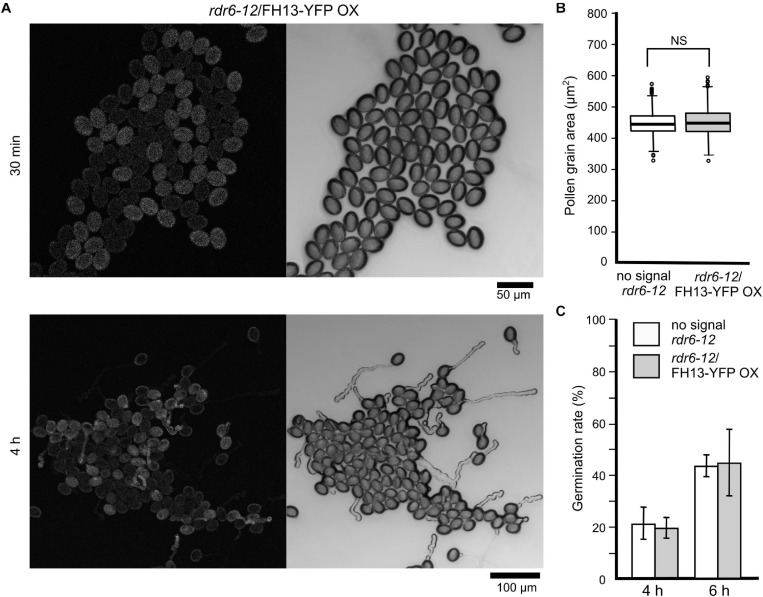
Pollen grain size and germination rate of the *rdr6-12*/FH13-YFP OX transgenic overexpression line **(A)** Representative confocal maximum intensity fluorescence projection (left) and single bright field optical section (right) of pollen from a *rdr6-12* plant heterozygous for the FH13-YFP OX transgene after 30 min and 4 h of culture. Note the difference between the fluorescent (transgenic) and non-fluorescent (non-transgenic) pollen grains. **(B)** Comparison of pollen grain area of non-transgenic (no signal) and *rdr6-12*/FH13-YFP OX (fluorescent) pollen grains at 30 min after plating (*n* > 300); NS, non-significant difference (one-way ANOVA, Tukey test *p* > 0.05). **(C)** Fraction of germinated pollen grains after the indicated time of *in vitro* cultivation. For genotype description see **(B)**; error bars represent ± SD from three technical replicates. Difference between transgenic and non-transgenic pollens at each of the time points were non-significant (χ^2^ test, *p* > 0.05).

To ensure high-level stable expression and easy detection of tagged FH13, we constructed transgenic plants expressing a FH13-YFP (yellow fluorescent protein) fusion protein under the control of the ubiquitin 10 (UBQ) promoter in the silencing-deficient *rdr6-12* background. Although the UBQ promoter is considered as a constitutive promoter active at moderate to strong level in all tissues (Grefen et al., 2010), no fluorescent signal was detected in any vegetative organs examined, while relatively strong fluorescence was observed in transgenic pollen grains and pollen tubes under standard *in vitro* culture conditions (Figure 5A). Based on the strong fluorescence signal in pollen and higher transcript level in vegetative tissues compared to transgenic plants expressing the same construct from the native FH13 promoter (see below), we consider this transgenic line an overexpressor, further referred to as *rdr6-12*/FH13-YFP OX.

To examine FH13 localization at native-like expression levels, we also constructed transgenic plants expressing a FH13-Venus fusion protein under the control of the native *FH13* promoter in both WT and homozygous *fh13-1* backgrounds, further referred to as WT/FH13-Venus and *fh13-1*/FH13-Venus, respectively. Similar to *rdr6-12*/FH13-YFP OX, the florescent protein signal was detected only in pollen grains and pollen tubes of these lines (Supplementary Figure S1A).

**FIGURE 6:**
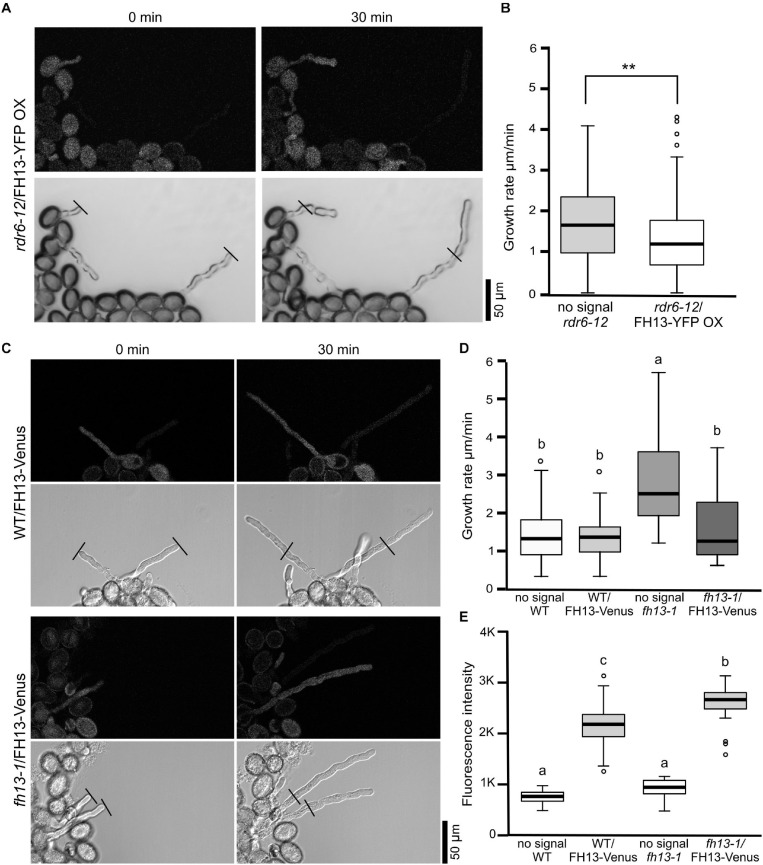
*In vitro* pollen tube growth of transgenic lines. **(A)** Representative single optical sections of pollen tubes from an *rdr6-12* plant heterozygous for the FH13-YFP OX transgene, taken at a 30 min interval 4 h after sowing on solid medium. Fluorescence (top) and bright field (bottom) channels are shown in grayscale. Note the presence of both fluorescent (i.e., transgenic) and non-fluorescent (non-transgenic) meiotic segregants in the same field. **(B)** Growth rates of non-transgenic (no signal) and FH13-YFP OX *rdr6-12* (fluorescent) pollen tubes (*n* > 40) from the line shown in **(A)**. Asterisks indicate a statistically significant difference (one-way ANOVA, Tukey test, ** for *p* < 0.01). **(C)** Representative single optical sections of pollen tubes from transgenic plants derived either from WT (top) or *fh13-1* (bottom) background and heterozygous for the FH13-Venus transgene, taken at a 30 min interval 4 h after sowing on solid medium. Fluorescence (top) and bright field (bottom) channels are shown in grayscale, presenting both transgenic and non-transgenic meiotic segregants. **(D)** Growth rates of non-transgenic (no signal) and FH13-Venus (fluorescent) pollen tubes from plant lines with either WT of *fh13-1* genetic background (*n* > 20). **(E)** Fluorescence intensities of FH13-Venus in WT and *fh13-1* mutant backgrounds (signal) and controls (no signal) measured from single optical sections of pollen grains (in arbitrary units, *n* > 30). Significant differences in **(D,E)** are marked by different letters (one-way ANOVA, Tukey test, *p* < 0.01).

In pollen cultures from plants carrying any of the transgenes in a heterozygous state, transformed and non-transformed pollen grains were readily distinguishable by fluorescence, allowing thus direct evaluation of the transgene’s effect on various male gametophyte parameters using non-transgenic (non-fluorescent) sister meiotic segregants as within-sample controls. Neither the FH13-YFP OX transgene nor the native promoter-driven constructs affected pollen grain size (Figure 5B and Supplementary Figure S1B) or germination rate (Figure 5C and Supplementary Figure S1C) in any of the genetic backgrounds examined. However, *rdr6-12*/FH13-YFP OX pollen tubes grew remarkably more slowly than non-transgenic *rdr6-12* controls (Figure 6A), and the inhibition of pollen tube growth by overexpression of tagged FH13—i.e., a phenotype opposite to that of loss of function mutants—was confirmed also by quantitative measurements (Figure 6B). On the other hand, pollen tubes expressing tagged FH13 from the native promoter were shorter than non-transgenic ones only in the *fh13-1* background, while no effect was observed in the WT background (Figure 6C). Quantitative growth rate analyses confirmed that native-level expression of FH13-Venus does not affect the growth of WT pollen tubes but fully restores the growth rate of *fh13-1* mutant pollen tubes to values indistinguishable from the WT (Figure 6D).

To investigate whether the presence of endogenous WT FH13 affected the level of FH13-Venus expression, we compared fluorescence intensities in transgenic WT and *fh13-1* pollen grains. The results indicated a significant, though not large, increase in transgene expression level in the *fh13-1* mutant background compared to WT pollen (Figure 6E). Additionally, we used semi-quantitative RT-PCR with specific primers to detect the 3′ portion of the FH13 transcript in young seedlings. The results revealed substantially higher transcript levels in transgenic lines compared to control WT plants and confirmed absence of full-length FH13 transcript in the *fh13-1* mutant, as well as a very high transcript level in the *rdr6-12*/FH13-YFP OX plants (Supplementary Figure S2). This finding is somewhat surprising given the absence of visible fluorescent protein signal in vegetative tissues including seedlings of the transgenic plants, and suggests possible post-transcriptional regulation of tagged FH13 protein level. Thus, in spite of the apparent mRNA overexpression in seedlings, the tagged protein may be present in our WT/FH13-Venus and *fh13-1*/FH13-Venus at a near-native levels, but may be overexpressed in the *rdr6-12*/FH13-YFP OX line.

These observations document that moderate level expression of Venus-tagged FH13 is sufficient to complement the effect of the *fh13-1* loss of function mutation, demonstrating that the original mutant is recessive, and that FH13 is responsible for the altered pollen tube growth rate. At the same time, high-level expression of YFP-tagged FH13 elicits a phenotype opposite to loss of function *fh13* mutations. Together, these data show that our fluorescent protein-tagged FH13 derivatives are biologically active.

### FH13 Exhibits Non-homogenous Cytoplasmic Localization in Pollen Tubes

**FIGURE 7:**
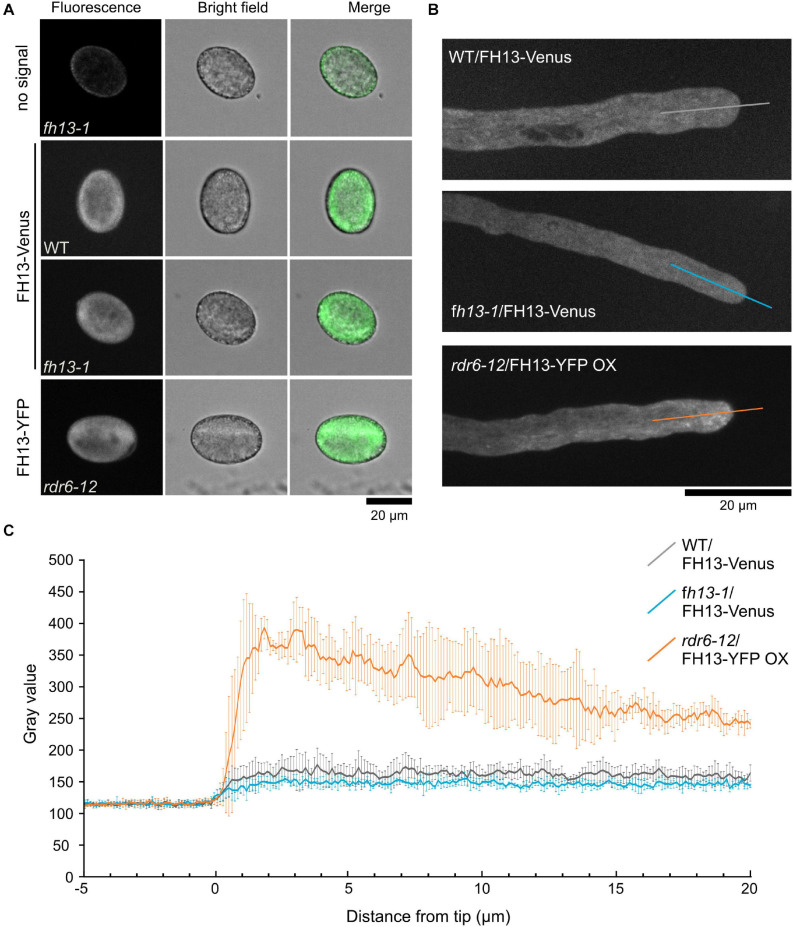
Localization of fluorescent protein-tagged FH13 in pollen grains and pollen tubes. **(A)** Representative single optical sections of control and transgenic pollen grains of the indicated genotypes. **(B)** Representative optical section of transgenic pollen tubes of the indicated genotypes, with transects used to construct fluorescence intensity plots shown. **(C)** Fluorescence intensity plots along transects shown in **(B)**, average intensity values (in arbitrary units) from four pollen tubes per genotype shown. Error bars represent SD.

To determine intracellular localization of FH13, we observed pollen grains and pollen tubes of transgenic WT/FH13-Venus, *fh13-1*/FH13-Venus and *rdr6-12*/FH13-YFP OX lines using spinning disc confocal microscopy. In mature hydrated pollen grains of all three lines the fluorescent protein signal displays similar pattern, suggesting cytoplasmic distribution with apparent exclusion from the central vacuole (Figure 7A). In growing pollen tubes, the FH13-Venus signal was cytoplasmic (Figure 7B and Supplementary Videos S1, S2) with similar intensity in tip and shank region (Figure 7C). On the other hand, the fluorescent signal in FH13-YFP OX pollen tubes, while still present in the cytoplasm, exhibited obvious enrichment at the tube tip (Figures 7B,C and Supplementary video S3). Mobile punctate and fibrous structures were clearly distinguishable in the very pollen tube tips (Figure 7B and Supplementary video S3), although it is not clear whether these brighter objects, whose distribution in some pollen tubes resembles an inverted cone pattern, represent some naturally present intracytoplasmic structures or protein aggregates arising as a consequence of labeled FH13 overexpression.

### Loss of FH13 May Affect Actin Organization in Pollen Tubes

**FIGURE 8:**
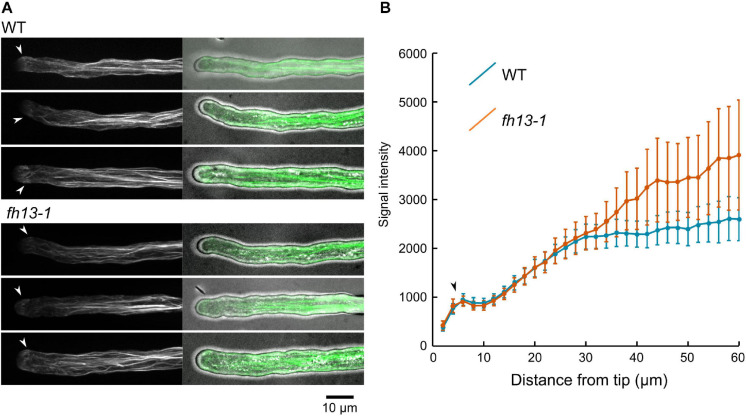
Organization of LifeAct-GFP-tagged microfilaments in WT and *fh13-1* pollen tubes. **(A)** Representative maximal intensity projections of confocal Z-stacks of transgenic pollen tubes of the indicated genotypes. **(B)** Averages of F-actin signal intensity values in ROIs spaced along the pollen tubes (in arbitrary units). Values (in arbitrary units) from 15 to 20 pollen tubes per genotype are shown. Error bars represent SE. Arrowheads in **(A,B)** mark position of the microfilament fringes.

Since formins are well-established as actin organizers, we next examined the structure and dynamics of the actin cytoskeleton in pollen tubes of homozygous WT or *fh13-1* plants expressing the LifeAct-GFP filamentous actin marker by live microscopy imaging. Both genotypes exhibited qualitatively normal microfilament organization characterized by an F-actin-free zone at the tip, followed by a noticeable “fringe” of fine microfilaments and a shank containing increasingly prominent microfilament bundles with growing distance from the tube tip (Figure 8A). In both genotypes, microfilaments were highly dynamic (Supplementary videos S4, S5). Since visual inspection of primary microscopy data suggested possible inter-genotype differences in the intensity of F-actin bundles signal along the shank of the pollen tube, we measured overall F-actin-related fluorescence intensity along the length of the pollen tube in both genotypes Although the observed difference between WT and *fh13-1* tubes was not statistically significant, we noticed a consistent tendency toward increased amount of bundled actin along the shank of mutant pollen tubes (Figure 8B). It is therefore possible that differences in actin organization may, at least in part, be responsible for the observed effects of *fh13* mutations on pollen tube growth.

## Discussion

This study focuses on the role of the *Arabidopsis* Class II formin FH13, reported to be expressed in pollen (Pina et al., 2005; Blanchoin and Staiger, 2010; Mergner et al., 2020), in the male gametophyte. To our knowledge, this is the first report addressing the role of a canonical Class II formin in pollen of a dicotyledonous plant, although a rice member of the same formin clade, FH5/RMD, was reported to contribute to achieving normal pollen tube growth rate and therefore proposed as a component of the gametophytic tip growth machinery (Li et al., 2018).

To elucidate the relationship between *Arabidopsis* FH13 and the previously characterized rice FH5, we first performed a detailed multi-domain phylogenetic revision of representative canonical Class II formins from twelve plant species. Previous phylogenetic analyses involving angiosperm Class II formins were either single-domain studies, usually based on the well-conserved FH2 domain, resulting in poor statistical support for internal branches of the Class II formin clade (Cvrčková et al., 2004; Rivero et al., 2005; Grunt et al., 2008), phylogenies focusing on other aspects of formin evolution and therefore incompletely sampled (Liu et al., 2010) or studies restricted to Brassicaceae (Cvrčková et al., 2012; Kollárová et al., 2020). Nevertheless, their results are largely consistent with existence of three distinct Class II subfamilies corresponding to the FH13/18, FH14, and FH20 clades identified in the present phylogenetic analysis (Figure 1), which was based on combined PTEN, C2 and FH2 domain sequences (the fourth domain ubiquitous in Class II formins, FH1, being highly repetitive and variable in length, could not be meaningfully aligned and was thus excluded from the analysis). Notably, since the only Class II formin with an established function in angiosperm pollen tubes, rice FH5 or RMD (Li et al., 2018) belongs to the FH20 clade, i.e., a different subfamily than *Arabidopsis* FH13, these two formins can be expected to have distinct biological functions. None of the three subclades, however, can be considered orthologous to any of the previously described *P. patens* formins, since moss Class II formins, also documented to participate in tip growth (Vidali et al., 2009; van Gisbergen et al., 2012, 2020), reliably cluster together and outside the angiosperm lineage (Grunt et al., 2008).

Based on available transcriptome data, all *Arabidopsis* canonical Class II formins are moderately expressed both in vegetative tissues and in pollen, and also the expression patterns reported from other species do not suggest the existence of a clade with a pollen-specific expression pattern (Figure 1 and Supplementary Table S1). This is in contrast with the Class I formin clade, whose branch Ic appears to contain predominantly pollen-expressed paralogs with tip growth-related roles (Cheung et al., 2010; Li et al., 2017; Lan et al., 2018). Nevertheless, earlier reports based on limited number of transcriptome datasets suggested pollen- and root hair-specific expression of *Arabidopsis FH13* (Blanchoin and Staiger, 2010; Schoenaers et al., 2017). *FH13* has even been reported as a gene expressed selectively in pollen (Pina et al., 2005), although expression levels under the limit of reliable detection were found in vegetative tissues in the same study. In an earlier report (Honys and Twell, 2003) *FH13* was not picked up as a pollen-expressed gene. Results of our mining of publicly available gene expression data, confirmed by semiquantitative RT-PCR assays (Figure 2), suggest that while *FH13* mRNA is present in pollen at a considerable level, it is by no means expressed exclusively in the male gametophyte. We suspect that the reported pollen specificity might be ultimately traceable to an artifact of the assay and data analysis algorithm used in the Pina et al. (2005) study. Nevertheless, *FH13* expression in pollen, supported also by successful pollen expression of a fluorescent protein-tagged transgene driven by the native *FH13* promoter (Supplementary Figure S1), is relatively high and clearly suggests a biological role of this gene in the male gametophyte.

Consistent with widespread FH13 expression, a recent pioneering proteomic study (Mergner et al., 2020) found both the *FH13* transcript and peptides derived from FH13 at detectable levels in a wide variety of tissues, with highest relative abundance in pollen (although differences between pollen and vegetative tissues were all within one order of magnitude). It is therefore somewhat surprising that we observed detectable fluorescence of C-terminally fluorescent protein-tagged FH13 only in the pollen, but not in vegetative tissues, of transgenic plants expressing FH13-Venus or FH13-YFP under the control of either the endogenous *FH13* promoter or of the supposedly constitutive Ubiquitin 10 promoter, respectively (Grefen et al., 2010; Figure 5 and Supplementary Figure S1), Fluorescent protein signal was absent even in young seedlings, where abundant mRNA, apparently originating from the transgene, was detected by RT-PCR (Supplementary Figure S2). Since this expression pattern was seen in independently transformed plant lines, it is unlikely to be due to a positional effect of transgene insertion. We rather suspect a tissue-specific difference in our fusion protein expression efficiency, resulting in its abundance not copying that of its transcript, and possibly of native FH13. A possible reason might be removal of the endogenous *FH13* 3′ UTR during construction of our expression vectors. Indeed, 3′ UTRs are known to contribute to transcript stability and therefore also translation efficiency of multiple plant transcripts (see Srivastava et al., 2018). While our observation opens some interesting questions regarding post-transcriptional regulation of FH13 expression, here we are focusing on the biological role and intracellular localization of this formin in pollen, where we have achieved satisfactory expression of the labeled protein.

Initial characterization of two mutant alleles, *fh13-1* and *fh13-2*, carrying T-DNA insertions inside the *FH13* locus, has been performed. Homozygous mutant plants are viable, free of readily noticeable developmental alterations and fully fertile. Both alleles can be efficiently transmitted by either male or female gametophytes, and their transmission ratios did not significantly deviate from the Mendelian F2 generation ratio of 1:2:1, although we noticed some under-representation of WT progeny that might perhaps be related to the subsequently observed faster growth of *fh13* pollen tubes (Table 1). Nevertheless, expression of full-length FH13 is obviously dispensable for grossly normal sporophytic and gametophytic development. This is in good agreement with studies of other single mutants in Arabidopsis (or other angiosperm) formins, which often exhibit subtle, if any, phenotypic alterations due to functional redundancy among the numerous members of the formin family, many of them with at least partially overlapping expression patterns (e.g., Cheung et al., 2010; Rosero et al., 2013; Lan et al., 2018). Remarkably, a rare case of a formin loss-of-function mutants with a readily noticeable phenotype involves the rice canonical Class II formin FH5 that was independently identified in two forward screens based on a mutant phenotype of dwarf, misshapen (yet viable and fertile) plants (Yang et al., 2011; Zhang et al., 2011). Since FH5 is the only rice FH20 clade member, these observations hint at possible functional diversification among Class II formin subfamilies.

As this study focuses on the male gametophytic development, we concentrated on characterizing the effect of *fh13* mutations on pollen structure, pollen germination and pollen tube development. The only phenotypic alteration consistently observed for two independent T-DNA alleles was significantly increased *in vitro* pollen tube growth rate compared to WT pollen, resulting in consistently greater pollen tube length in mutant *in vitro* pollen cultures (Figure 4). Small but significant increase in pollen grain size was, in addition, found only in the *fh13-2* line (Figure 3). It should be, however, noted that WT segregants from a backcrossed heterozygous *fh13-1* plant were used as WT control, and that the *fh13-2* line (which has not been extensively backcrossed), although derived from the same T-DNA mutagenesis experiment, could carry some additional subtle differences in its genetic background.

Homozygous mutant plants carrying *fh13-1* or *fh13-2* lack the full length *FH13* mRNA but might express a small amount of a truncated transcript (Figure 2), leaving the possibility that the increased pollen tube growth rate might be due to the presence of a partial gene product rather than to the absence of the WT protein, analogous e.g., to the case of truncated *Arabidopsis* FH8 inhibiting root hair development (Deeks et al., 2005). We consider this possibility unlikely because we succeeded in restoring normal growth rate of *fh13-1* pollen tubes by expression of fluorescent protein-tagged full length FH13 (Figure 6), indicating that the *fh13-1* mutation is recessive.

Plant formins have been so far viewed in the context of tip growth mainly as organizers of the actin cytoskeleton, whose behavior is crucial for proper pollen tube growth (reviewed in Zhang et al., 2018). Arabidopsis Class I formins FH3 and FH5 are responsible for polymerization of membrane-originated actin cables at the pollen tube tip, thus facilitating and maintaining tip growth (Ye et al., 2009; Cheung et al., 2010; Lan et al., 2018). The rice Class II formin FH5 is also localized to the pollen tube apex; mutants in the FH5/RMD gene exhibit decreased pollen tube elongation rate and misshapen, twisting or turning pollen tubes caused by defective actin structures critical for tip-focused growth (Li et al., 2018). Low-level heterologous expression of *Arabidopsis* FH1, a sporophytically expressed Class I formin, stimulated tobacco pollen tube growth, while massive FH1 overexpression in the same system resulted in pollen tube growth arrest and tube tip swelling, most likely due to disruption of the normal balance in actin dynamics and the overall actin cytoskeleton structure (Cheung and Wu, 2004). Indeed, depletion of several profilin isoforms results in reduced pollen tube growth, as well as pollen tube thickening and twisting suggestive of a partial loss of polarity (Liu et al., 2015), consistent with the profilin to formin ratio contributing to determination of tube tip growth rate. Formins in general, and Class II formins in particular, are thus mainly viewed as proteins acting in the angiosperm tip growth machinery as positive effectors or positive regulators whose loss leads to growth suppression.

Surprisingly, loss of function *fh13* mutants exhibit significantly faster pollen tube elongation than WT plants, which can be reverted by a close-to-native level expression of a fluorescent protein-tagged FH13, while moderate FH13-YFP overexpression inhibit pollen tube growth *in vitro* (Figure 6). A similar phenotype of enhanced pollen tube growth has been reported for loss of function mutations of genes affecting cytoskeletal, especially actin, organization. The pollen-expressed MDA25 (Microtubule-destabilizing protein 25) destabilizes also microfilaments through its F-actin severing activity in the subapical cytoplasm of pollen tubes and its loss leads to enhanced pollen tube growth, surprisingly associated with decreased fertilization efficiency due to defective pollen tube guidance (Qin et al., 2014). Another negative regulator of pollen tube growth, RIC1 (a ROP-interactive CRIB motif-containing protein) has been found to control the abundance and dynamics of microfilaments by severing and capping activity at the apical plasma membrane as well as in the cytoplasm (Zhou et al., 2015). Since some formins exhibit not only actin nucleation and capping, but also actin severing activities (see Courtemanche, 2018), we could speculate that such an activity of FH13 might be responsible for its observed ability to inhibit pollen tube elongation. However, an actin-severing activity has not yet been reported in a plant formin, and may even be a specific feature of a subset of metazoan formins. Nevertheless, our observations suggesting a possible increase in the amount of microfilament bundles in *fh13* pollen tubes (Figure 8) are consistent with alterations in actin cytoskeleton organization contributing to the observed phenotype.

Another explanation may be based on analogy with recent observations in the moss *P. patens*. The moss Class II formin For2 is found at the tip of apically growing cells, localizes to endomembrane structures in a manner dependent on its PTEN domain (Vidali et al., 2009; van Gisbergen et al., 2012), and has been recently proposed to participate in endocytosis of excess membrane exocytosed during rapid tip growth (van Gisbergen et al., 2020). The exocytosis—endocytosis balance is an important factor in controlling the rate of tip growth. Its disruption may result in increased pollen tube growth rate, as observed e.g., in *Arabidopsis* mutants lacking the negative exocytosis regulator EXO70C2, which also exhibit compromised cell wall integrity and frequent recoverable bursting of the pollen tube tip (Synek et al., 2017). The observed non-homogenous cytoplasmic localization of fluorescent protein-tagged FH13 (Figure 7) appears to be consistent with such a role in membrane trafficking. In the apical cytoplasm of some pollen tubes overexpressing FH13-YFP, the protein exhibits remarkable accumulation of punctate and fibrous structures in a pattern reminiscent of the endomembrane-rich “inverted cone” region characterized not only by bulk exocytotic vesicle flow (Hepler and Winship, 2015) but probably also by endocytotic activity (Grebnev et al., 2017). Localization of overexpressed FH13-YFP also somewhat reminds of that of EXO70C2 and its relative EXO70C1 (Synek et al., 2017), previously found to interact with the negative exocytosis regulator ROH1 (Kulich et al., 2010). However, the observed pattern in overexpressing pollen tubes is also consistent with a simple gradient in cytoplasmic fluorescent protein abundancy, previously reported e.g., for GFP-tagged monomeric G-actin (Chang et al., 2017), and observations from pollen tubes with lower transgene expression levels support cytoplasmic localization of FH13 rather than its association with specific intracellular structures.

In summary, we hypothesize that FH13, as the first experimentally addressed canonical Class II formin of the FH13/18 clade, may act as a negative modulator of pollen tube growth due to its participation in the control of cytoskeletal dynamics, but possibly also related to some function(s) affecting membrane trafficking.

## Data Availability Statement

The raw data supporting the conclusions of this article will be made available by the authors, without undue reservation.

## Author Contributions

EK and FC contributed to the conception and design of the study, prepared the figures, and wrote the first draft of the manuscript. EK and AB performed the experiments and evaluated experimental data. FC performed the bioinformatic analyses. All authors contributed to manuscript revision, read, and approved the submitted version.

## Conflict of Interest

The authors declare that the research was conducted in the absence of any commercial or financial relationships that could be construed as a potential conflict of interest.

## Footnotes

1 http://signal.salk.edu/tdnaprimers.2.html

## Supplementary Material

Supplementary Figure S1 Pollen grain size and germination rate of FH13-Venus transgenic lines **(A)** Representative confocal maximum intensity fluorescence projection (left) and single bright field optical section (right) of pollen from transgenic plants derived either from WT (top) or *fh13-1* (bottom) background and heterozygous for the FH13-Venus transgene after 30 min and 4 h of culture. **(B)** Comparison of pollen grain area of non-transgenic (no signal) and FH13-Venus transgenic (fluorescent) pollen grains from WT or *fh13-1* background plants at 30 min after plating (*n* > 250); NS, non-significant difference (one-way ANOVA, Tukey test *p* > 0.05). **(C)** Fraction of germinated pollen grains after the indicated time of *in vitro* cultivation. For genotype description see **(B)**; error bars represent ± SD from three technical replicates. Differences between transgenic and non-transgenic pollens at each of the timepoints were non-significant (χ^2^ test, *p* > 0.05).

Supplementary Figure S2 Detection of 3′ portions of the *FH13* transcript in WT/FH13-Venus, *fh13-1*/FH13-Venus and *rdr6-12*/FH13-YFP OX transgenic and WT, *fh13-1* and *rdr6-12* control 7 DAG seedlings by semiquantitative RT-PCR, with a fragment of the *UBQ* gene amplified as a control. The reactions were run for 24 and 28 cycles for *FH13* and at 26 cycles for *UBQ*. The arrowheads represent DNA ladder size (white-100 bp; light gray-200 bp; dark gray-300 bp). gDNA, genomic DNA; L, DNA ladder.

Supplementary Table S1 Inventory of canonical Class II formin sequences from twelve angiosperm species. For genes with available transcriptomic data, information on sources of expression data and on aboveground tissues or organs with highest transcript levels is provided.

Supplementary Table S2 Primers used for genotyping, cloning and semiquantitative RT-PCR.

Supplementary Video S1 Time lapse video of a growing pollen tube expressing FH13-Venus in WT background. An overlay of fluorescence (green) and bright field images is shown. Scale bar is 10 μm.

Supplementary Video S2 Time lapse video of a growing pollen tube expressing FH13-Venus in *fh13-1* background. An overlay of fluorescence (green) and bright field images is shown. Scale bar is 10 μm.

Supplementary Video S3 Time lapse video of a growing pollen tube overexpressing FH13-YFP in *rdr6-12* background. An overlay of fluorescence (green) and bright field images is shown. Scale bar is 10 μm.

Supplementary Video S4 Time lapse video of a growing pollen tube expressing LifeAct-GFP in WT background. The fluorescence channel is shown. Scale bar is 10 μm.

Supplementary Video S5 Time lapse video of a growing pollen tube expressing LifeAct-GFP in *fh13-1* background. The fluorescence channel is shown. Scale bar is 10 μm.
