# Supplementary material for: The Arabidopsis thaliana Class II Formin FH13 Modulates Pollen Tube Growth
Source: Front Plant Sci. 2021 Feb 18;12:599961. doi: 10.3389/fpls.2021.599961 (PMC7929981; doi:10.3389/fpls.2021.599961)
Supplement: Supplementary Figure S1 — Pollen grain size and germination rate of FH13-Venus transgenic lines (A) Representative confocal maximum intensity fluorescence projection (left) and single bright field optical section (right) of pollen from transgenic plants derived either from WT (top) or fh13-1 (bottom) background and heterozygous for the FH13-Venus transgene after 30 min and 4 h of culture. (B) Comparison of pollen grain area of non-transgenic (no signal) and FH13-Venus transgenic (fluorescent) pollen grains from WT or fh13-1 background plants at 30 min after plating (n > 250); NS, non-significant difference (one-way ANOVA, Tukey test p > 0.05). (C) Fraction of germinated pollen grains after the indicated time of in vitro cultivation. For genotype description see (B); error bars represent ± SD from three technical replicates. Differences between transgenic and non-transgenic pollens at each of the timepoints were non-significant (χ2 test, p > 0.05). [file Image_1.PDF]

Supplementary Material – Kollárová et al.

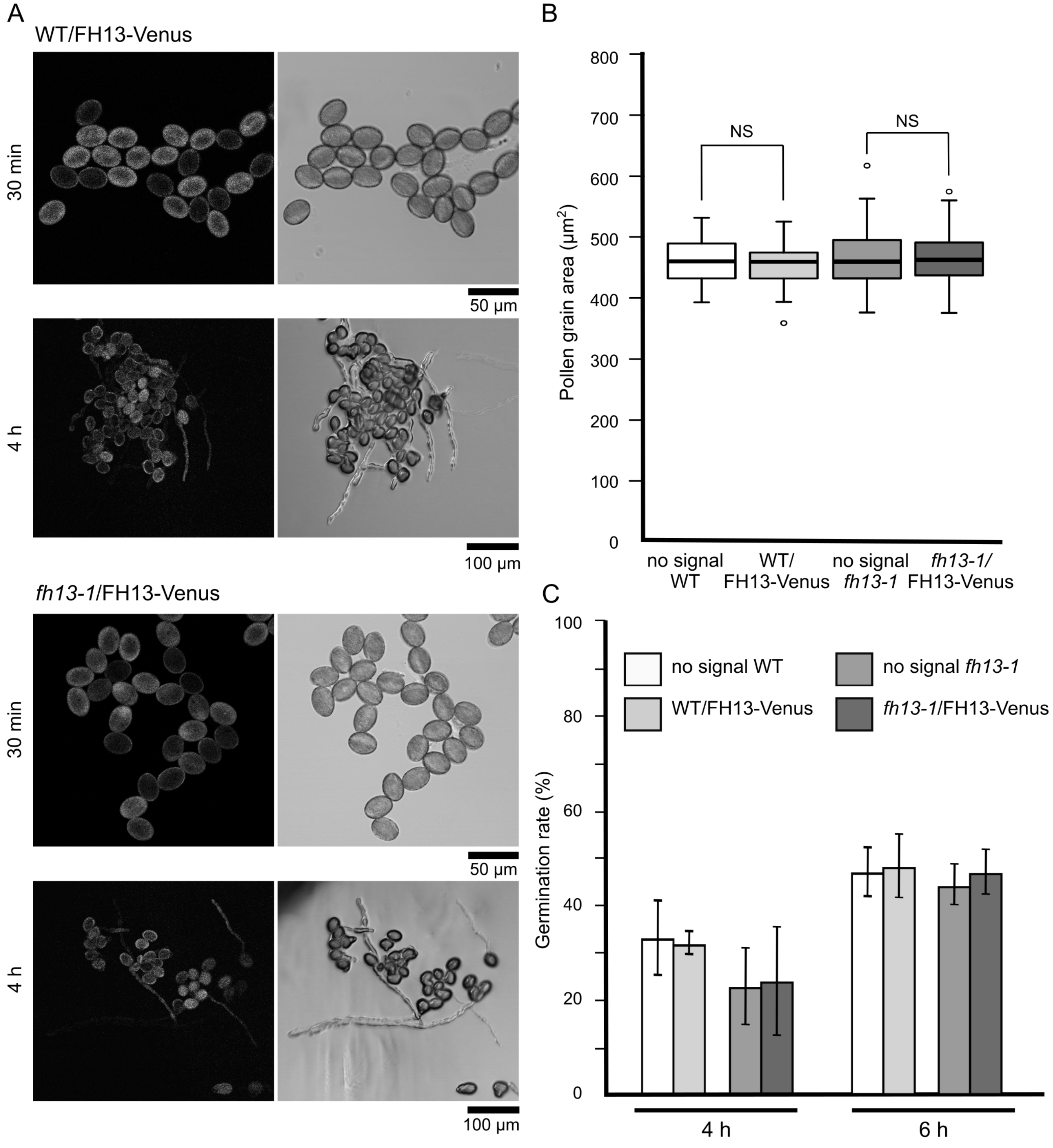

Supplementary Figure S1. Pollen grain size and germination rate of FH13-Venus transgenic lines (A) Representative confocal maximum intensity fluorescence projection (left) and single bright field optical section (right) of pollen from transgenic plants derived either from WT (top) or *fh13-1* (bottom) background and heterozygous for the FH13-Venus transgene after 30 min and 4 h of culture. (B) Comparison of pollen grain area of non-transgenic (no signal) and FH13-Venus transgenic (fluorescent) pollen grains from WT or *fh13-1* background plants at 30 min after plating (n> 250); NS, non-significant difference (one-way ANOVA, Tukey test p>0.05). (C) Fraction of germinated pollen grains after the indicated time of *in vitro* cultivation. For genotype description see (B); error bars represent ± SD from three technical replicates. Difference between transgenic and non-transgenic pollens at each of the timepoints were non-significant (χ^2 test, p>0.05).
