# Supplementary material for: The Arabidopsis thaliana Class II Formin FH13 Modulates Pollen Tube Growth
Source: Front Plant Sci. 2021 Feb 18;12:599961. doi: 10.3389/fpls.2021.599961 (PMC7929981; doi:10.3389/fpls.2021.599961)
Supplement: Supplementary Figure S2 — Detection of 3′ portions of the FH13 transcript in WT/FH13-Venus, fh13-1/FH13-Venus and rdr6-12/FH13-YFP OX transgenic and WT, fh13-1 and rdr6-12 control 7 DAG seedlings by semiquantitative RT-PCR, with a fragment of the UBQ gene amplified as a control. The reactions were run for 24 and 28 cycles for FH13 and at 26 cycles for UBQ. The arrowheads represent DNA ladder size (white-100 bp; light gray-200 bp; dark gray-300 bp). gDNA, genomic DNA; L, DNA ladder. [file Image_2.PDF]

Supplementary Material – Kollárová et al.

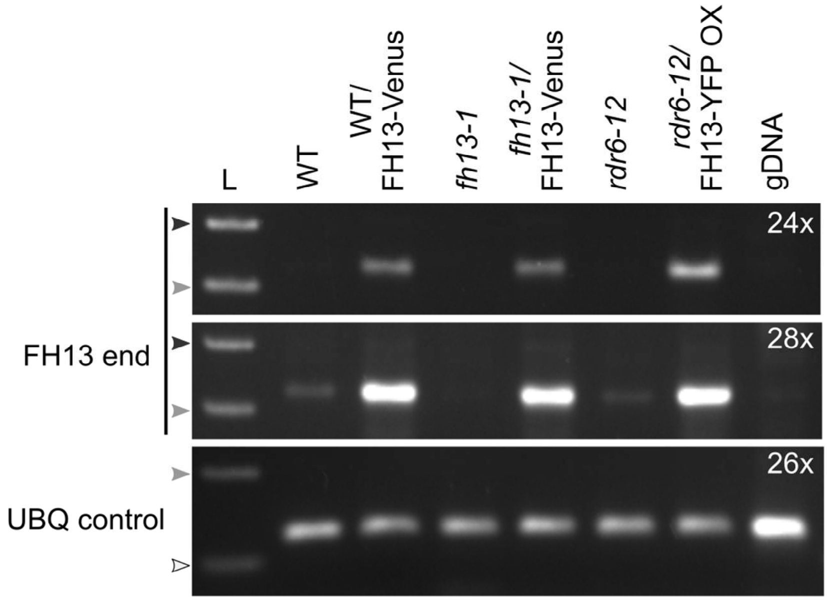

Supplementary Figure S2. Detection of 3´portions of the *FH13* transcript in WT/FH13-Venus, *fh13-1*/FH13-Venus and *rdr6-12*/FH13-YFP OX transgenic and WT, *fh13-1* and *rdr6-12* control 7 DAG seedlings by semiquantitative RT-PCR, with a fragment of the *UBQ* gene amplified as a control. The reactions were run for 24 and 28 cycles for *FH13* and at 26 cycles for *UBQ*. The arrowheads represent DNA ladder size (white -100bp; light gray-200bp; dark gray-300bp). gDNA – genomic DNA; L – DNA ladder.
