# Supplementary material for: The Arabidopsis thaliana Class II Formin FH13 Modulates Pollen Tube Growth
Source: Front Plant Sci. 2021 Feb 18;12:599961. doi: 10.3389/fpls.2021.599961 (PMC7929981; doi:10.3389/fpls.2021.599961)
Supplement: Supplementary Table S2 — Primers used for genotyping, cloning and semiquantitative RT-PCR. [file Table_2.PDF]

Supplementary Material – Kollárová et al.

Supplementary Table S2. Primers used for genotyping, cloning and semiquantitative RT-PCR.

Primer	Sequence 5' - 3'	Use
FH13_314_LP	GTGTGTTGCAGTGTTCGATTG	Genotyping
FH13_314_RP	ACCACTCGAAACATCATGACC	
FH13_291_RP_new	TCCAAGAAGGACTAGATGGTA	
FH13_291_LP	TTTGACCTAATGGTGGTGGAG	
SALK LBb1.3	ATTTTGCCGATTTCGGAAC	
pAtFH13_for	TGCTTGTGAATTCTACGGAC	Cloning
pAtFH13_rev	CTTCGAATTTCAAAAATCGACAAC	
RT_AtFH13_for5end	AGATGATGAAAGTCCCACGAG	RT-PCR
RT_AtFH13_rev5end	TTAAACCCAACCGCAGCAC	
RT_AtFH13_for6start	TCGCCCTTGAACCCAATTCC	
RT_AtFH13_rev6start	CATTCTGCCTGCTTGTAGACC	
